# Supplementary figures and images for: Seasonal variations in social contact patterns in a rural population in north India: Implications for pandemic control
Source: PLoS One. 2024 Feb 22;19(2):e0296483. doi: 10.1371/journal.pone.0296483 (PMC10883557; doi:10.1371/journal.pone.0296483)

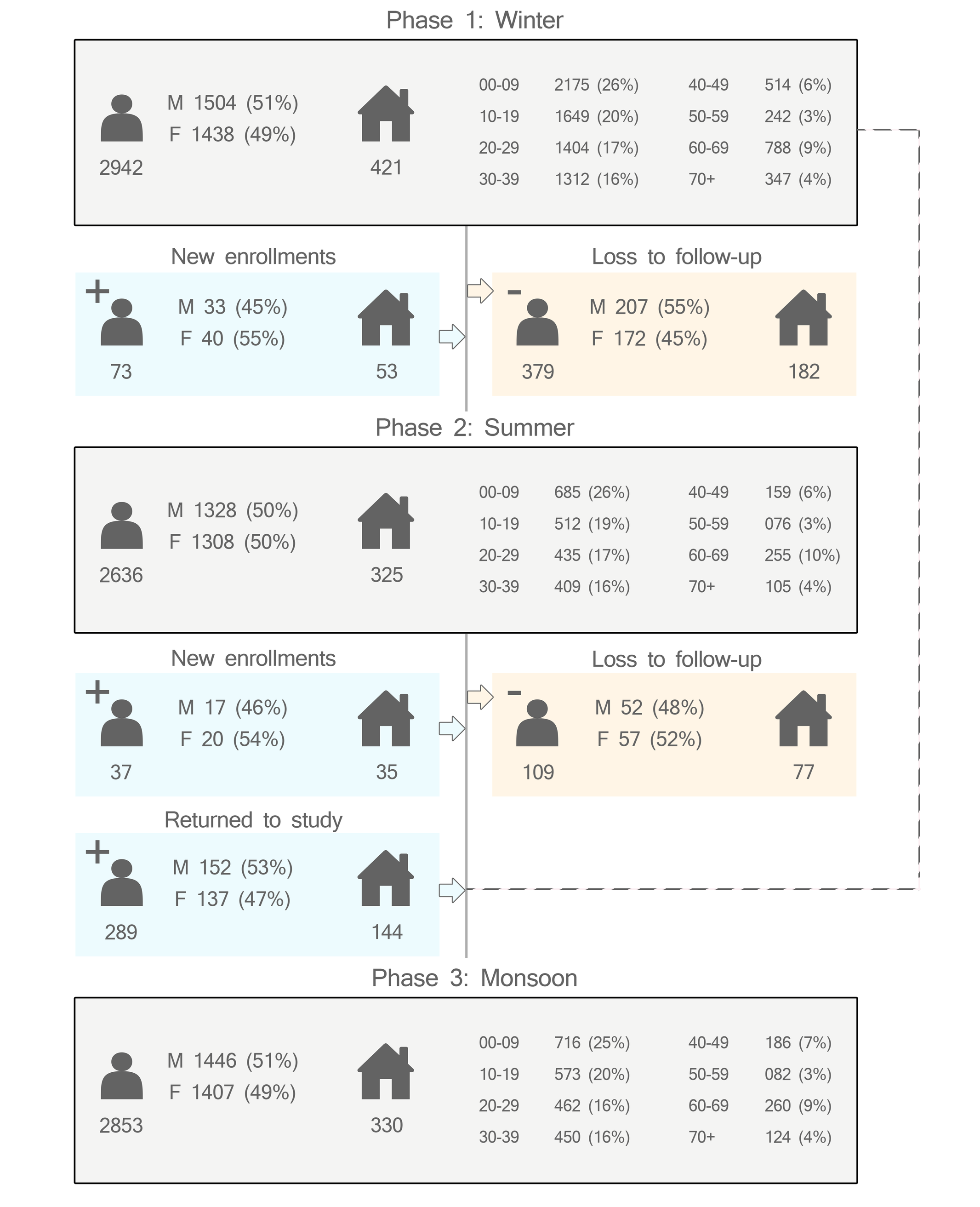

Supplement: S1 Fig — The age and gender stratified respondent counts per season are displayed in the grey blocks, while the blue and orange blocks show the loss and gain of respondents in follow-up surveys. (TIF) [file pone.0296483.s001.tif]

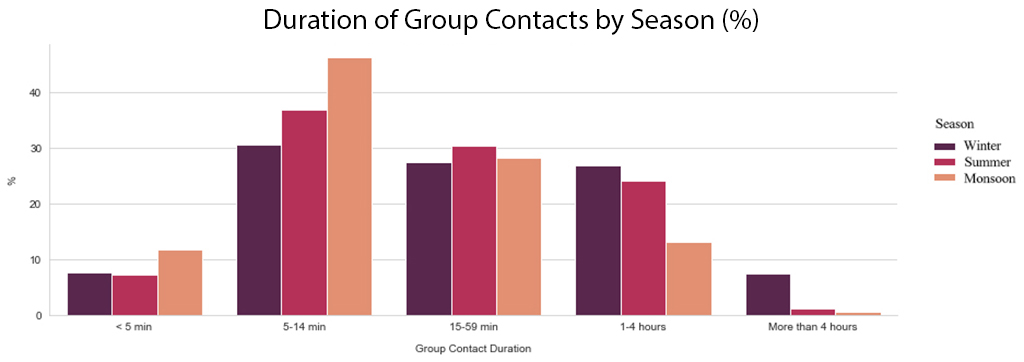

Supplement: S2 Fig — Winter had a higher percentage of group contacts longer than 4 hours, compared to the other waves. (TIF) [file pone.0296483.s002.tif]

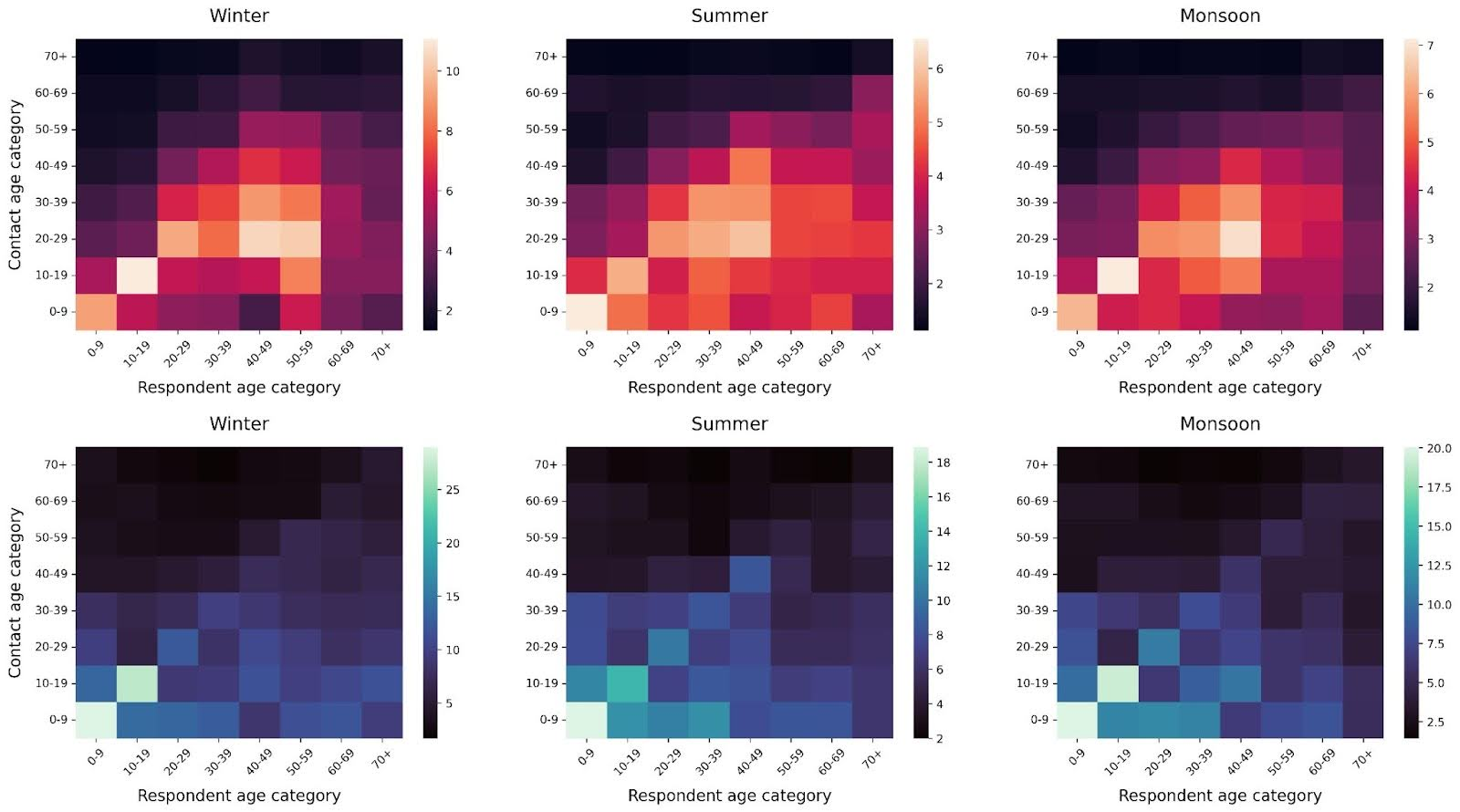

Supplement: S3 Fig — Heatmaps representing mean numbers (red) and durations (blue, in person-hours) of contacts reported between age category dyads. Brighter colours imply a pair of categories with a high number/duration of contacts. Bright diagonal elements suggest some form of age-assortativity. (TIF) [file pone.0296483.s003.tif]

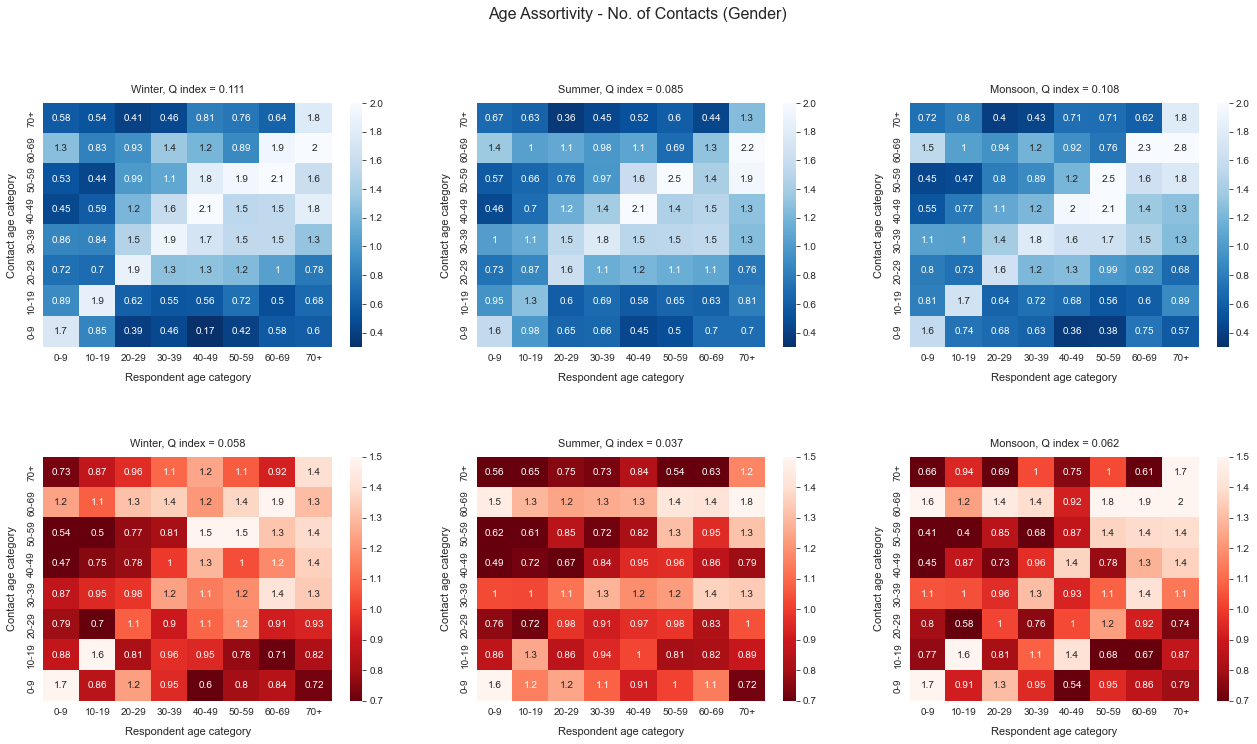

Supplement: S4 Fig — Gender-stratified heat-maps representing the average number of contacts reported between two age categories (Males in blue, females in red). Brighter colours imply a pair of categories with a high number of contacts. (TIF) [file pone.0296483.s004.tif]

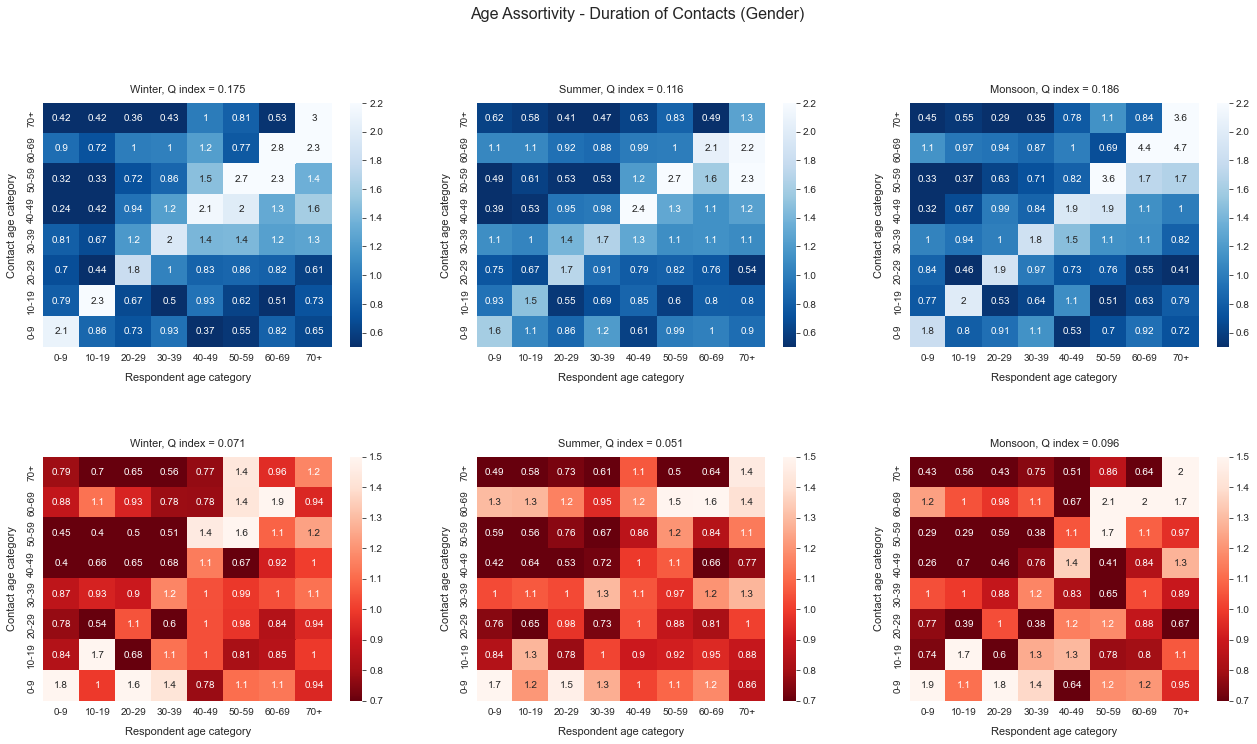

Supplement: S5 Fig — Gender-stratified matrices representing the average duration of contacts in person-hours reported between two age categories (Males in blue, females in red). Brighter colours imply a pair of categories with a high number of contacts. (TIF) [file pone.0296483.s005.tif]

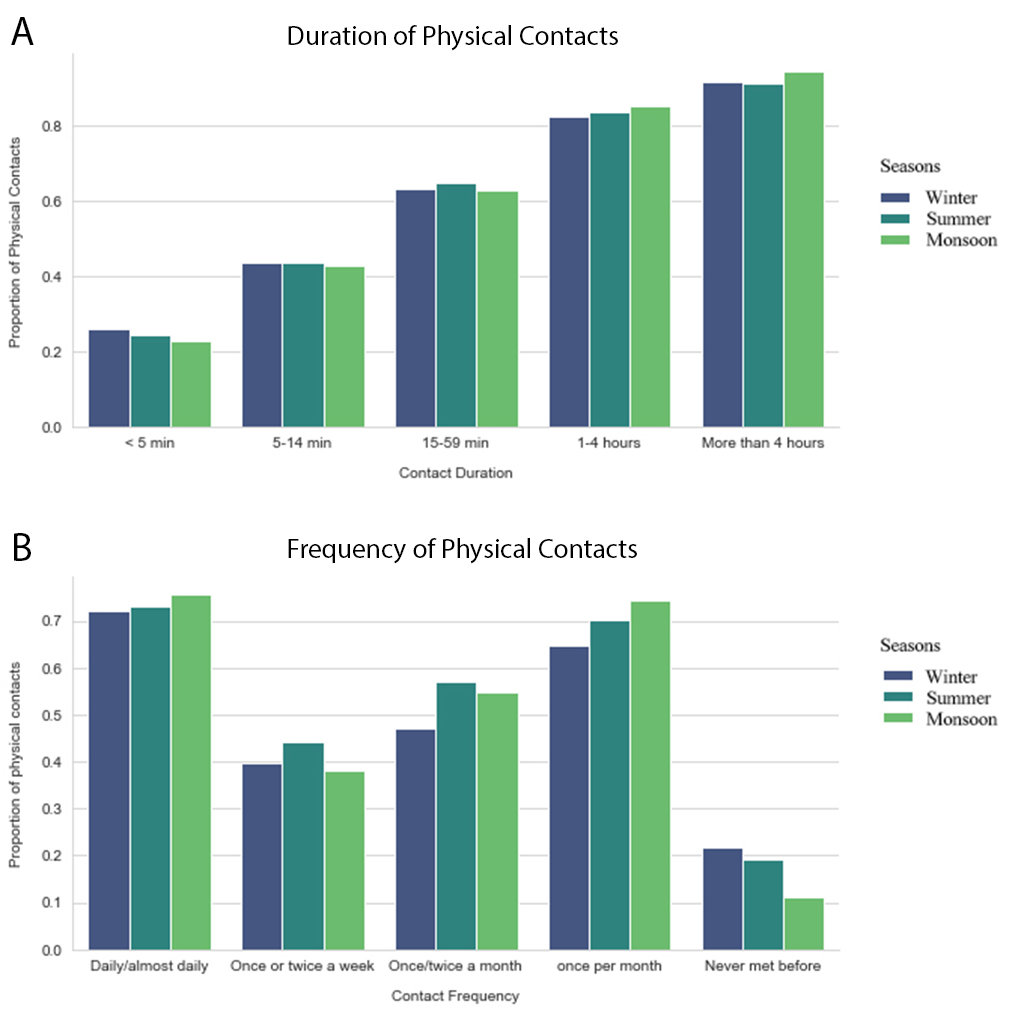

Supplement: S6 Fig — (A) Barplots of the proportion of contacts reported to involve physical touch for all three waves, stratified by the duration of the contact. (B) Proportion of physical contacts for all three waves stratified by the frequency of which the respondent met the contact. (TIF) [file pone.0296483.s006.tif]

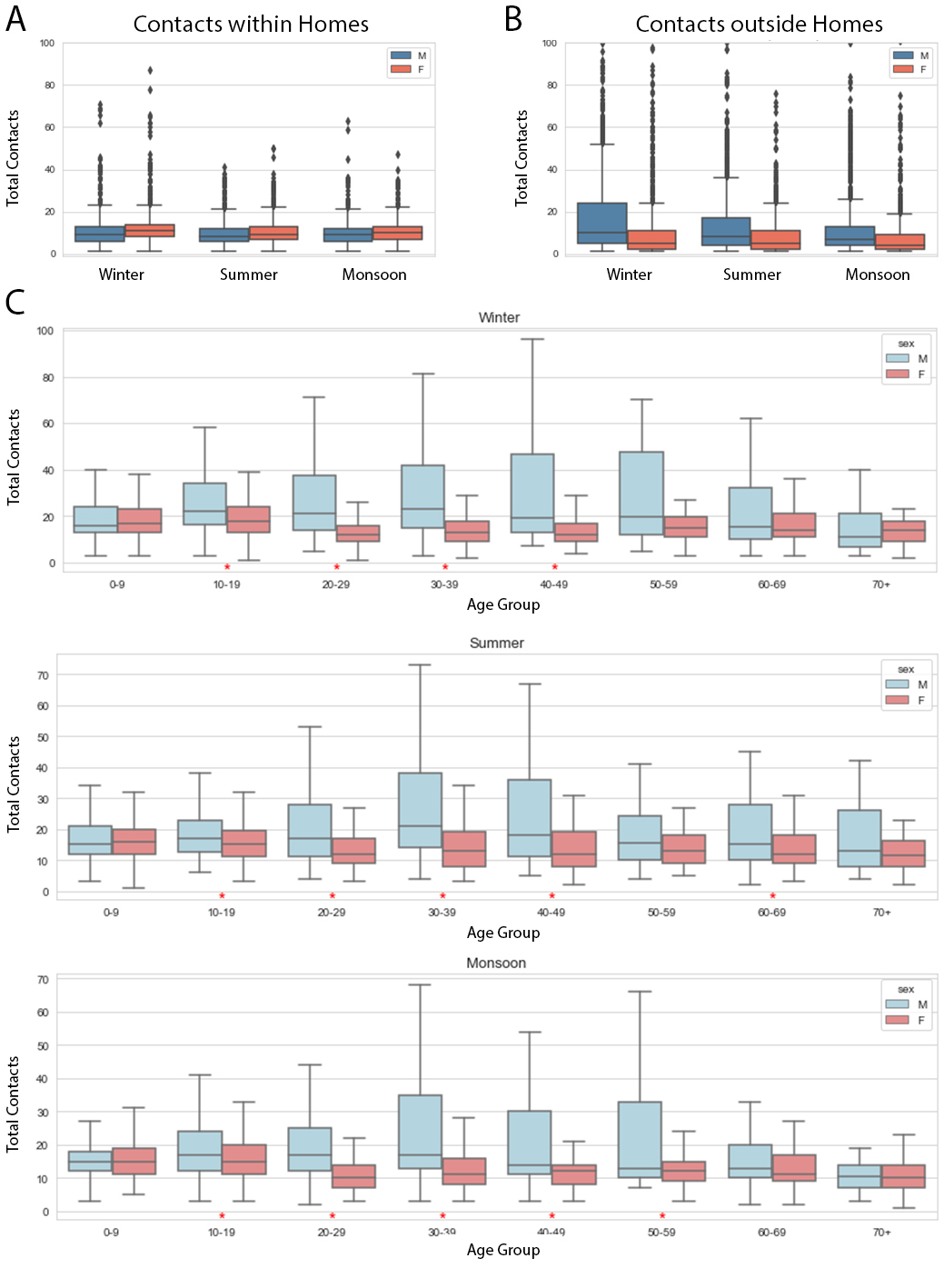

Supplement: S7 Fig — (A) Boxplots of the number of contacts that occurred at home reported by males (blue) and females (red). (B) Boxplots of the number of total contacts reported to have occurred outside the home. Note that the upper y limit has been truncated to match that of (A). (C) Boxplots of the number of contacts stratified by gender and age category, across every wave. (TIF) [file pone.0296483.s007.tif]

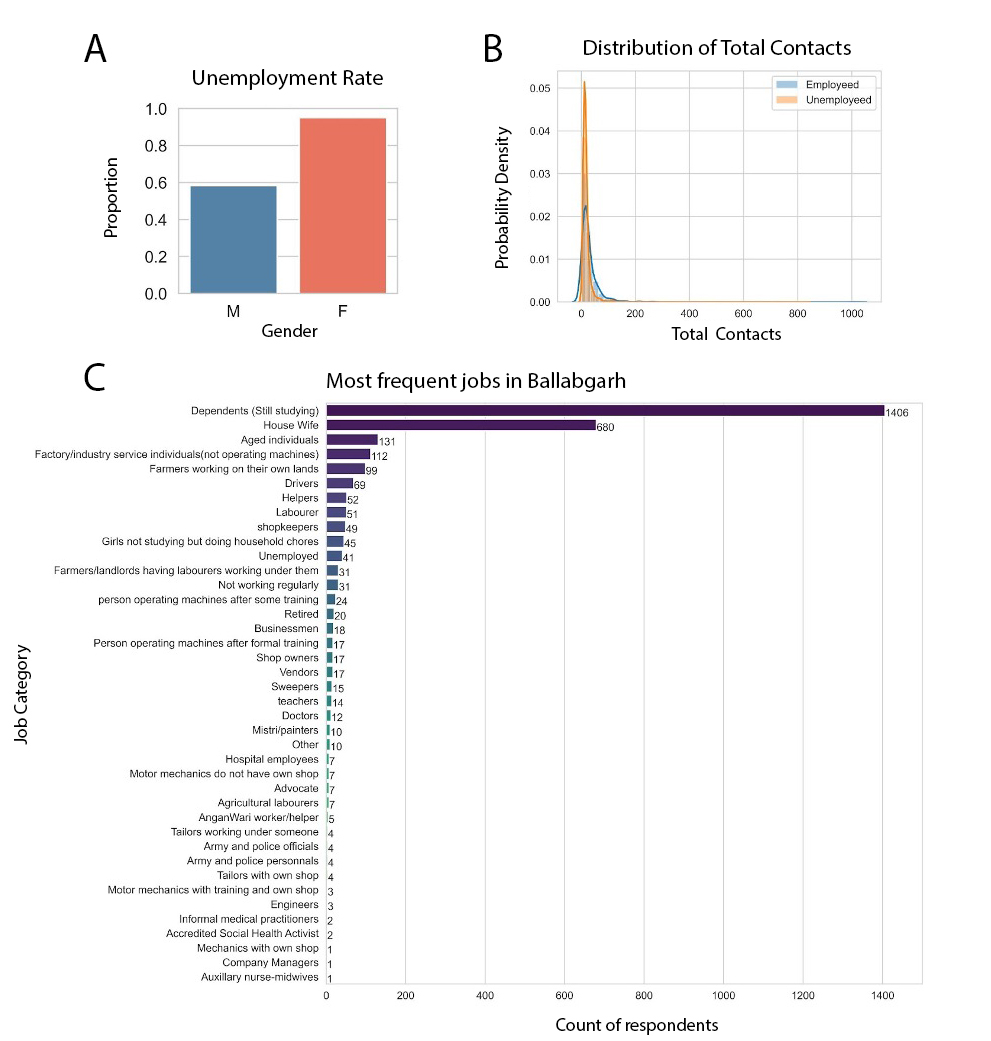

Supplement: S8 Fig — (A) Percentages of males and females classed as unemployed outside the home. (B) Distribution of total contacts for both employed and unemployed. Solid line represents a gaussian KDE. Note the longer tail on the distribution for employed respondents. (C) Frequencies of occupations among the respondents of the survey. (TIF) [file pone.0296483.s008.tif]

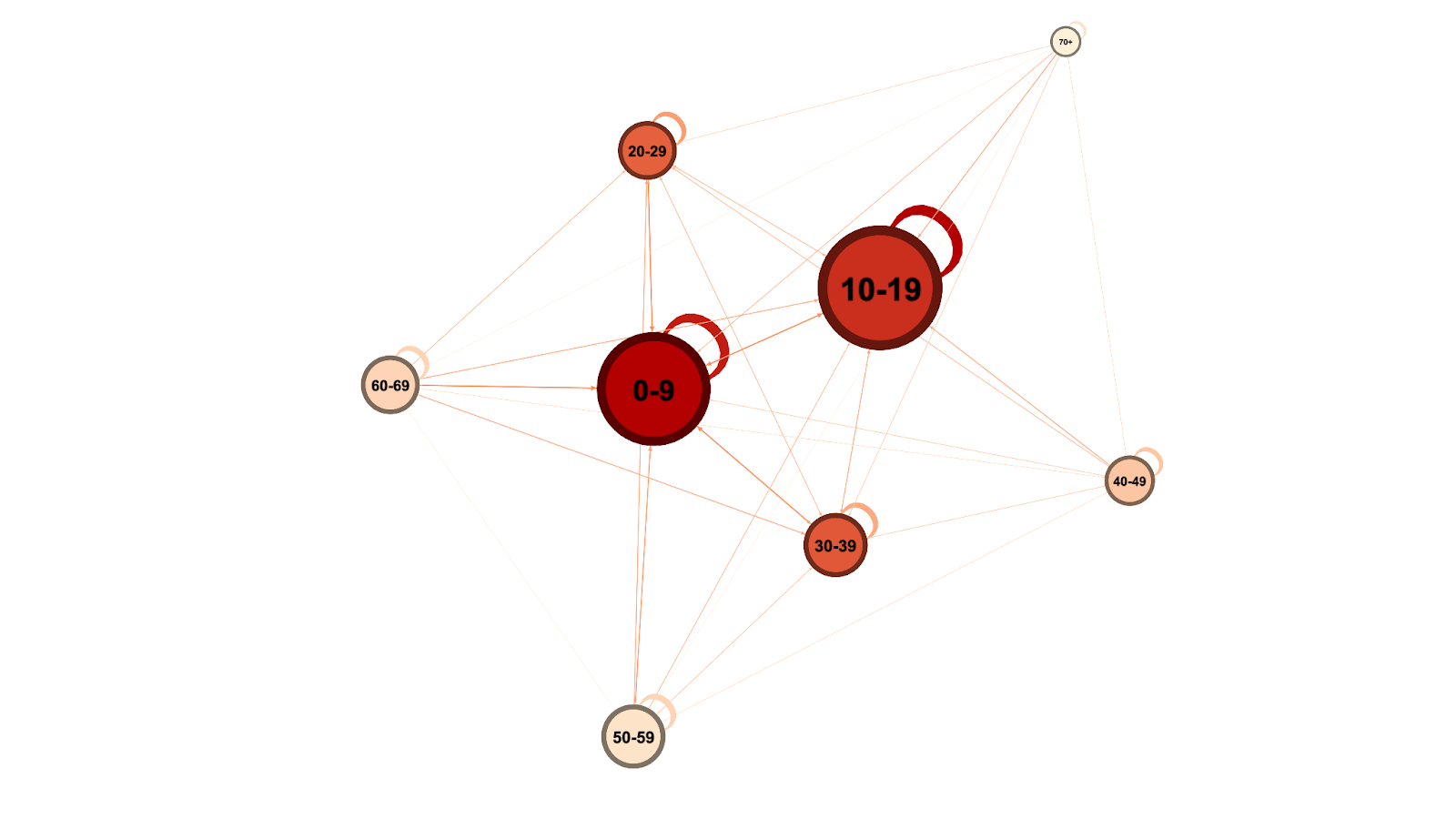

Supplement: S9 Fig — Directed graph representing the median number of contacts an age category has with every other age category. Higher numbers are depicted with stronger edge weights. Node sizes represent the total median contacts and node colours represent the PageRank of each node (darker = more important). (TIF) [file pone.0296483.s009.tif]

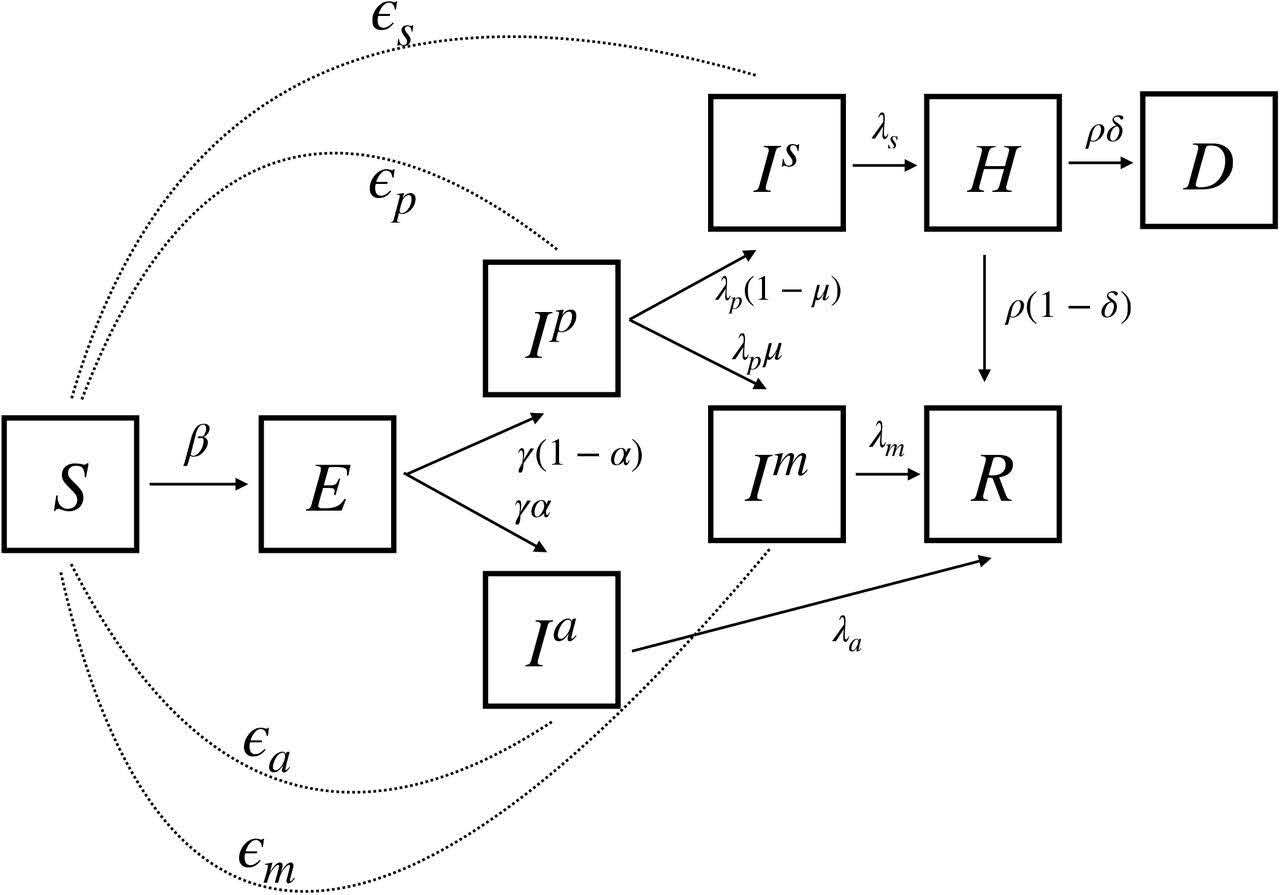

Supplement: S10 Fig — Figure sourced from Hazra et al.34 S = susceptible, E = exposed, Ip = Presymptomatic, Ia = Asymptomatic, Im = Mildly Infected, Is = Severely Infected, H = Hospitalised, R = Recovered, D = Deceased. (TIF) [file pone.0296483.s010.tif]

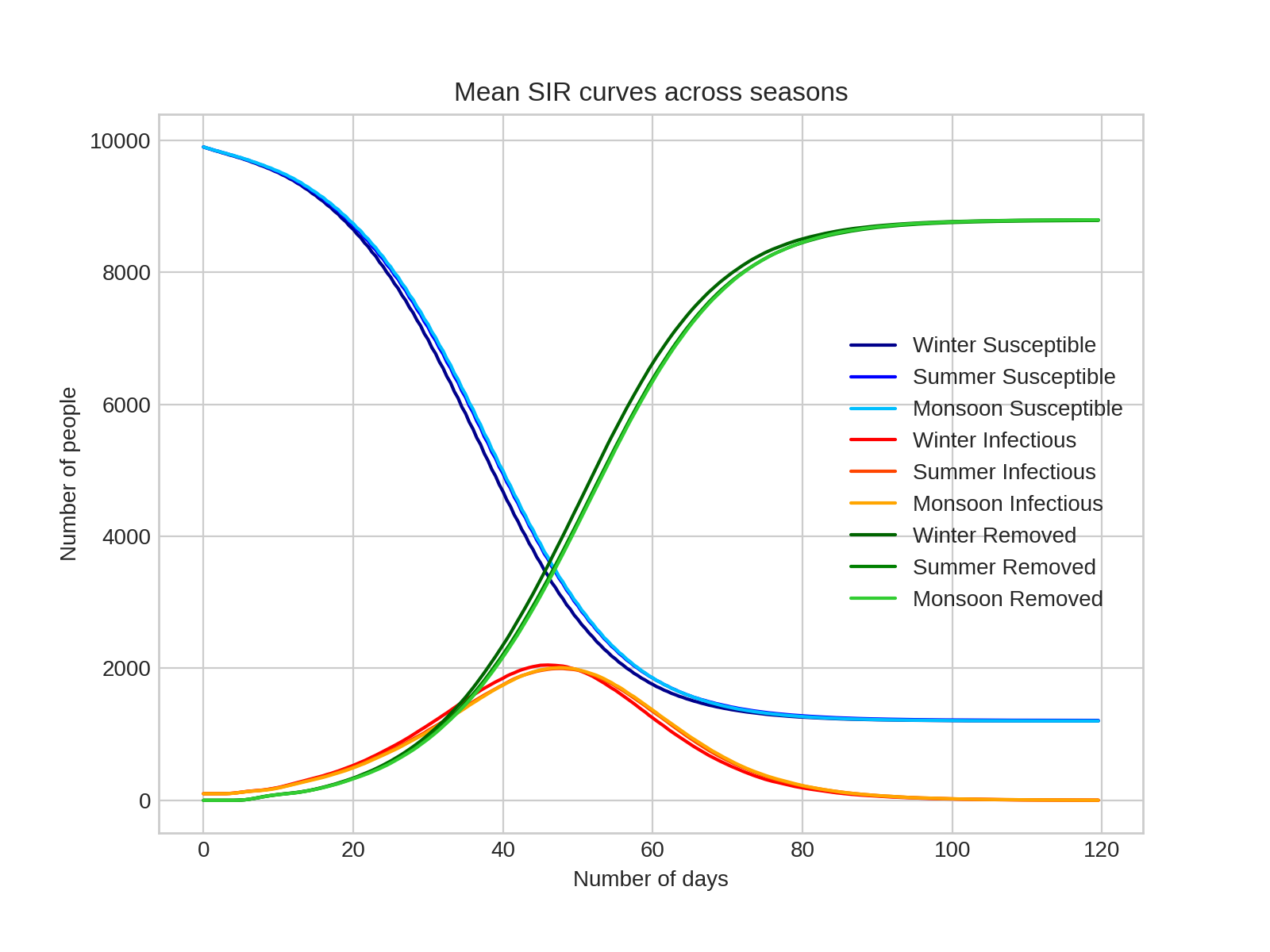

Supplement: S11 Fig — Blue, red and green represent susceptible, infectious, and removed counts respectively. Darker colours represent the winter, lighter colours monsoon and summer. Lines are the mean of 50 runs on a population of 10,000 for each season. (TIF) [file pone.0296483.s011.tif]

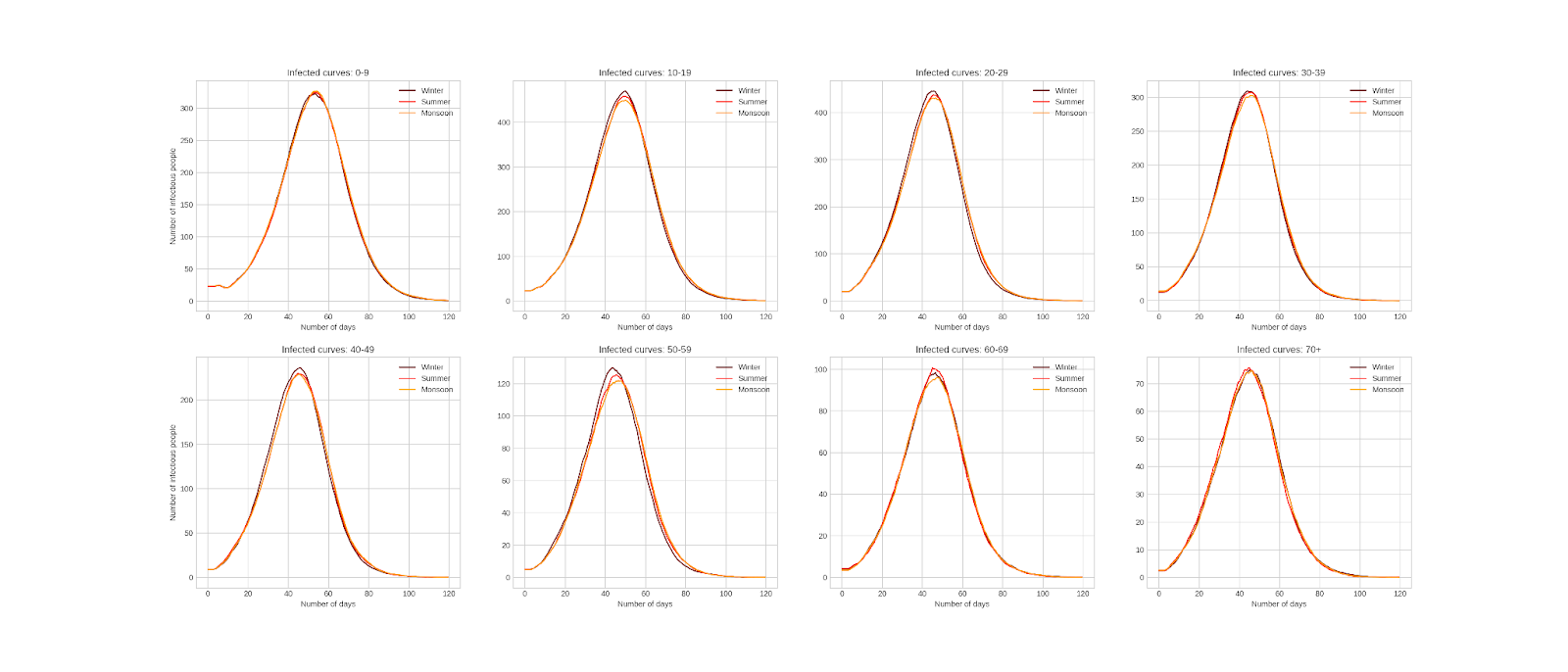

Supplement: S12 Fig — Mean number of infectious people across 50 simulations for every season who belong to a particular age category. Brown, red and orange represent winter, summer and monsoon seasons respectively. Significant difference across seasons is observed in the 50–59 category. (TIF) [file pone.0296483.s012.tif]

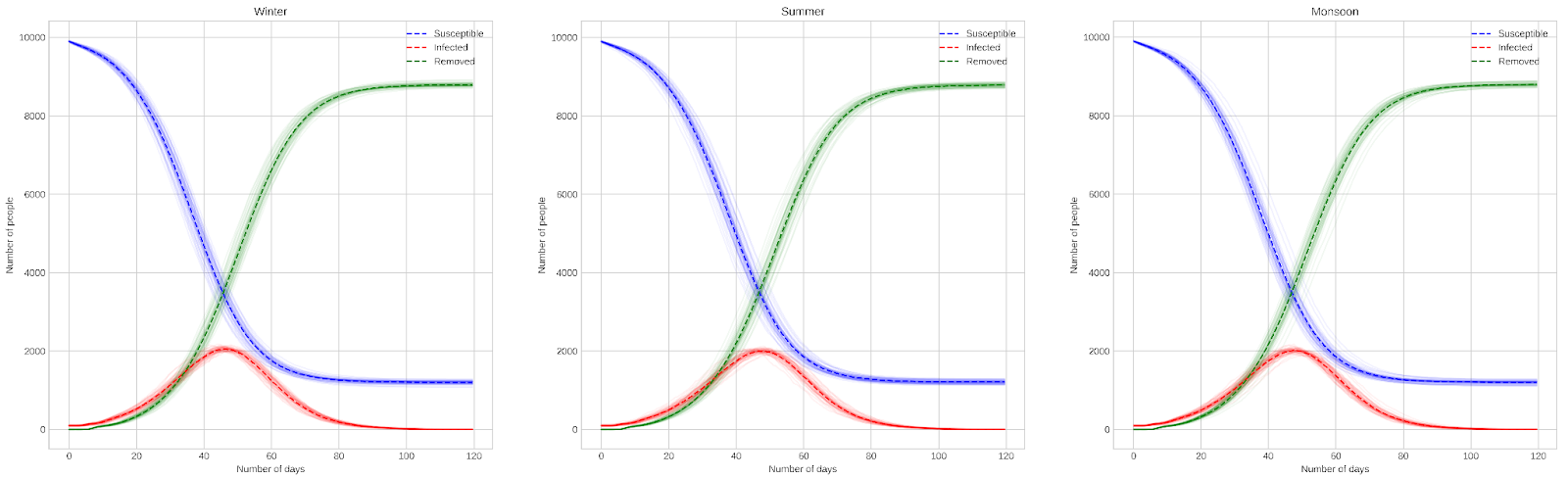

Supplement: S13 Fig — Blue, red and green represent susceptible, infectious, and removed counts respectively. Each solid line is data from a single run, dashed lines are the mean of 50 runs on a population of 10,000 for each season. (TIF) [file pone.0296483.s013.tif]

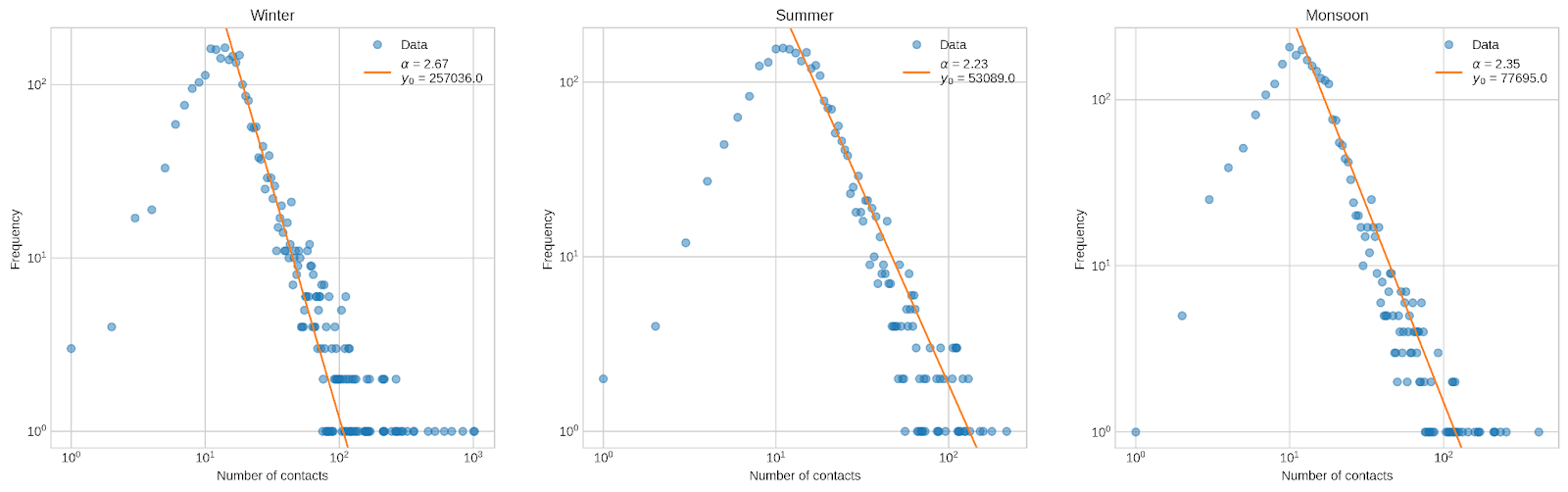

Supplement: S14 Fig — The number of individuals who reported a certain amount of total contacts is plotted against the number of total contacts. A curve of y=y0xα is fit on the descending section of the data. Note that ∝ represents the negative slope of the line on the log-log plot. (TIF) [file pone.0296483.s014.tif]
