# Supplementary material for: Seasonal variations in social contact patterns in a rural population in north India: Implications for pandemic control
Source: PLoS One. 2024 Feb 22;19(2):e0296483. doi: 10.1371/journal.pone.0296483 (PMC10883557; doi:10.1371/journal.pone.0296483)
Supplement: S1 Table — (PDF) [file pone.0296483.s015.pdf]

S.No	Attribute	Methodology
1	Dates	All date formats *(31/10/21, 31/10/2021, 31-Oct-21)* were normalised to a common date format. Manual correction of dates was performed based on the season (wave).
2	Respondent Age	The date of birth of each respondent and the date of interview was used to compute the age.
3	Contact Gender	Other mentions of the same name were filtered and the mode of the reported genders was used as the gender of the respondent. In case of conflicts, the Genderize API[2] was used to predict the gender based on the respondent name, followed by manual validation.
4	Group size	Imputed by the median group size of contacts occurring at the same location. For example, for a group contact at home with a missing group size, all group contacts at home were filtered and the median group size was used for imputation.
5	Duration of group contact	Imputed by the mode duration of group contact occurring at the same location.
6	Minimum, maximum age in group contact	Imputed by the median minimum and maximum ages of group contacts occurring at the same location.
7	Contact Age	Contact ages were imputed by sampling from the contact ages of similar respondents. Similar respondents were defined as people who had a similar contact age distribution based on their JS divergence. A Random Forest regression model was trained to predict the JS divergence between two respondents based on features such as their age difference, difference in the number and duration of contacts, proportion of contacts with males and more. A set of similar respondents was obtained for a given respondent and the missing contact ages were sampled from the contact age distribution of these respondents.
