# Supplementary material for: Seasonal variations in social contact patterns in a rural population in north India: Implications for pandemic control
Source: PLoS One. 2024 Feb 22;19(2):e0296483. doi: 10.1371/journal.pone.0296483 (PMC10883557; doi:10.1371/journal.pone.0296483)
Supplement: S2 Table — (PDF) [file pone.0296483.s016.pdf]

S.No	Group contact category	Keywords
1	Chatting	chating, chatting, chhatin
2	Death	condolence, death, derth, dearth, shok, sok
3	Festival	birthday, festival, holi, kanjikey, namkaran, raksha, rasam, sakrant
4	Game	cricket, game, kabbadi, play, sports
5	Madarsa	madarsa, madrasa, madatsra, madatrsa
6	Politics	chunav, election, rally, rss, vote, voter
7	School	aanganwari, aganwadi, anganwadi, anganwari, class, coaching, college, colledge, school, taleem, talim, tuition, tution
8	Shop	buy, dairy, dukan, market, milk, purchase, shoop, shop, vendor
9	Transport	auto, bus, ola, taxi, train, transport, travel
10	Wedding	barat, baraat, function, fuction, kanya, marriage, ring, sagai, sangeet, shadi, wedding
11	Work	business, company, duty, labour, office, job, polymed, wager, work
12	Worship	bhagvat, bhagwat, bhagwath, eid, jagran, jagrata, kirtan, langar, mandir, masjid, maszid, mosk, mosque, namaj, pray, ramayan, satsang, satssang, temple, worship
