# Supplementary material for: Seasonal variations in social contact patterns in a rural population in north India: Implications for pandemic control
Source: PLoS One. 2024 Feb 22;19(2):e0296483. doi: 10.1371/journal.pone.0296483 (PMC10883557; doi:10.1371/journal.pone.0296483)
Supplement: S3 Table — (PDF) [file pone.0296483.s017.pdf]

Month	chatting	death	festival	game	madarsa	other	politics	school	shop	transport	wedding	work	worship
Jan	290	255	270	119	744	833	4638	370	520	63	708	2458	110
Feb	123	150	0	85	185	556	0	606	987	665	2023	1735	95
Mar	77	215	216	97	100	397	0	358	839	0	294	160	192
Apr	174	140	0	82	40	605	0	110	183	28	625	506	167
May	110	180	25	158	135	490	0	354	575	258	422	1199	98
Jun	43	85	30	27	0	393	0	28	1012	315	20	2326	10
Jul	35	0	20	10	15	94	0	125	415	0	98	480	105
Aug	40	355	180	50	0	375	0	483	217	100	60	814	60
Sep	230	50	40	199	181	278	0	82	440	30	50	1123	153
Oct	190	350	50	130	70	1070	0	1064	1466	170	355	3716	285
Nov	90	100	30	131	0	1509	30	1108	1615	98	2200	354	688
Dec	90	312	0	7	350	1600	80	385	865	145	722	343	235
