# Supplementary material for: Seasonal variations in social contact patterns in a rural population in north India: Implications for pandemic control
Source: PLoS One. 2024 Feb 22;19(2):e0296483. doi: 10.1371/journal.pone.0296483 (PMC10883557; doi:10.1371/journal.pone.0296483)
Supplement: S5 Table — r_sus is relative susceptibility, and is multiplied to the value of a target’s beta. p_symp represents the probability of showing symptoms, p_severe the probability of displaying severe symptoms given the person is infected, and p_deceased the probability of death given the person has severe symptoms. Rates taken from the Covasim model developed by Kerr et al.33 (PDF) [file pone.0296483.s019.pdf]

	0-9	10-19	20-29	30-39	40-49	50-59	60-69	70-79	80-89	90+
r_sus[10]	0.34	0.67	1.00	1.00	1.00	1.00	1.00	1.24	1.47	1.47
p_symp[8,11]	0.50	0.55	0.60	0.65	0.70	0.75	0.80	0.85	0.90	0.90
p_severe[8,11]	0.005	0.00165	0.00720	0.02080	0.03430	0.07650	0.13280	0.20655	0.24570	0.24570
p_deceased[12,13]	0.00002	0.00002	0.00010	0.00032	0.00098	0.00265	0.00766	0.02439	0.08292	0.16190
