# Supplementary material for: Seasonal variations in social contact patterns in a rural population in north India: Implications for pandemic control
Source: PLoS One. 2024 Feb 22;19(2):e0296483. doi: 10.1371/journal.pone.0296483 (PMC10883557; doi:10.1371/journal.pone.0296483)
Supplement: Attachment — Submitted filename: Response to Reviewers.docx [file pone.0296483.s020.docx]

**Reviewer 1:**

Methods, Data cleaning. The authors mentioned that, in part, information related to the nature and frequency of group contacts was used to impute gender, where missing. Can the authors comment on how this may impact their inference/conclusions related to differences social mixing characteristics by gender?

Response: We thank the reviewer for the comment. The paper specifies the following:

“We further imputed contact genders by checking instances of the same name; the number, duration, and age range of group contacts by using the median of group contacts at the same location; and the contact ages by sampling from age distributions of similar respondents.”

We only used contact names to impute contact genders. We used information relating to group contacts to impute missing data on group contacts, such as the number of people in the group or the duration of the contact event. We observed that our data cleaning did not make any significant differences to our results

Methods, comparison of contacts … Can the authors clarify what is meant by “For this study, statistical methods for independent samples were used for comparison.”

Response: We thank the reviewer for the comment. We have added further clarification about statistical tests for independent and dependent samples.

“We performed two types of analyses of the data, the first being an analysis within each season, similar to the work of Kumar et al. [20], where we identified the differences in contact patterns of age groups for each season individually. For this study, statistical methods for independent samples were used for comparison (Kruskal Wallis, Dunn’s test) since each age category had distinct individuals, and these samples could be assumed independent. Further, we analysed the differences in contact patterns across the three seasons. For this study, participants who responded in two or more seasons were considered for analysis and statistical methods for dependent samples were used (Friedman, Nemenyi test) since the contacts of the same individual across multiple seasons cannot be assumed to be independent.”

Methods, Regression models to predict contacts and understand the effect of explanatory variables. Please provide more specificity with regard to how the negative binomial multivariate model(s) estimated the ‘independent effects’ of the characteristics listed. The reference to ‘hyperparameters’ suggests that you conducted these model fits from a Bayesian perspective. Is that correct, or am I reading too much into this statement?

Response: We thank the reviewer for the comment. The multivariate regression model measures the effect of each independent variable on the dependent variable (number of contacts) while controlling for other covariates in the model. We clarified this in the methods section. Furthermore, we would like to clarify that we did not perform any Bayesian hyperparameter search to optimize the fit of the regression model. We used the default function parameters from the ‘statsmodels’ Python package to fit the model.

“We also constructed a multivariate negative binomial regression model to understand the independent effect of the season, age-groups (in ten-year categories), employment, and whether the day was a weekend (while controlling for other covariates) on the number and duration of contacts.”

Methods, Generating a synthetic population that mimics contact patterns. The authors refer to the use of a Markov Chain Monte-Carlo (MCMC) approach to generate synthetic population(s) from, in part, the assortative mixing matrices derived from their contact survey data. Can the authors provide more details about the specifics of this MCMC approach? Typically, MCMC refers to an approach for randomly sampling chains of inter-related events (specifically, where the probability of the current sample being drawn depends solely on the value of the preceding sample in the chain). This type of procedure doesn’t seem conducive to generating synthetic populations. Possibly the authors are referring to Monte-Carlo approaches to generating synthetic populations?

Response: We thank the reviewer for the comment. We have clarified that we do indeed mean Monte-Carlo approaches in the Methods section.

“We generated a population of size 10,000 (one for each season) with an age distribution similar to that of the census data for Faridabad, and used a Monte-Carlo approach following the simulated annealing metaheuristic to assign every agent a house and a workplace, such that the age-stratified contact pattern in homes and workplaces resembled the observed pattern of contacts within the home and outside the home respectively.”

Results.

Since 95.4% of the respondents responded to surveys in more than one season, have the authors accounted for within-individual correlation in their analyses, for example, within the multivariable negative binomial models. Also, do the author’s hypothesis testing procedures for inter-season variability account for the very-high degree of participants responding in multiple seasons?

Response: We thank the reviewer for the question. We included the “season” as a covariate in the Negative Binomial model to adjust for the repeated measurements of the same individuals. Furthermore, for hypothesis testing, we treated the contacts of the same individual across multiple seasons as non-independent and used statistical tests for dependent samples (Friedman test, and Nemenyi post-hoc test). We have clarified this further in the methods section.

“Further, we analysed the differences in contact patterns across the three seasons. For this study, participants who responded in two or more seasons were considered for analysis and statistical methods for dependent samples were used (Friedman, Nemenyi test) since the contacts of the same individual across multiple seasons cannot be assumed to be independent.”

Figure 4. To aid in the interpretation of Figure 4, it might help to present the contact duration information on the hours-per-contact scale. It is hard to interpret and make use of information on the Total Duration scale, for example, to parameterize a stochastic individual-based simulator.

Response: We thank the reviewer for the suggestion. In our work, we aimed to study the aggregate-level statistics on the total number and duration of contacts across the seasons, stratified by age-groups and gender. For example, we aimed to answer questions like - “Which age group spends the most amount of time on average with 0-9 year old males?”. Such a group might be targeted to limit social contacts with 0-9 year males. To calculate this, we make use of the total duration of contacts, which is a function of both the duration of each individual contact and the number of such contacts with 0-9 year olds. If we quantify duration on the hours-per-contact scale, we fail to capture the total contact durations of the respondents.

Figure S10. Legend. The authors state in the text that they used a individual-based stochastic epidemic simulator to simulate SARS-CoV-2/COVID-19 epidemics in their synthetic population, but this figure’s legend refers to a ‘compartmental model’ (i.e., a system of differential equations). Did the authors use a system of differential equations or a stochastic individual-based simulator with the possible states shown in Figure S10? If the latter, can the authors provide some explanation as to why the curves shown in Figure S11 and S12 seem to be overly smoothed for the typical outcome of a stochastic individual-based simulator?

Response: We thank the reviewer for the comment. We use an agent based model, where every agent has a 'state' which represents which stage of the infection they're currently in. We have updated the legend of *Figure S10* to clarify the same. The curves shown in figures *S11* and *S12* are averaged over 50 simulations, which makes the stochasticity less apparent.

In addition, 50 simulations seem to be a bit low number of simulation to run for the complex system represented by this synthetic population. Can the authors provide some plot or description of the epidemic curves from the simulated epidemics to provide the reader with some description of the variability in the resulting simulated epidemics.

Response: We thank the reviewer for the comment. We have added a figure with the individual epidemic curves in *Figure S13* (and renamed the original *Figure S13* to *Figure S14*).

**Reviewer 2:**

Introduction:

1. The introduction could benefit from reference to systematic reviews of contact patterns (both for high/low income countries), for example: Hoang et al, 2019 and Mousa et al, 2021.

Response: We thank the reviewer for the comment. We have added the following line to the introduction:

“However, systematic reviews of contact patterns [[13]](https://paperpile.com/c/iJs9jG/psN0)[[14]](https://paperpile.com/c/iJs9jG/qWaQ) indicate a distinct lack of data from low-income countries”

2. The authors mention some of the studies which have attempted to measure and/or analyse temporal patterns. However, there is no summary of what were the main findings from these studies.

Response: We thank the reviewer for the comment. The investigative direction of the studies were varied: some, like Eames et al. (<https://doi.org/10.1371/journal.pcbi.1002425>) sought to compare contacts during the school term with those during school vacations. As we do not have data on school closures, this is not something we can emulate, and thus we decided to primarily focus on mentioning the studies which we can draw direct comparisons with.

A brief summary of the results of Béraud et al (<https://doi.org/10.1371/journal.pone.0133203>) is already present in the discussion. We have added the following line to the discussion mentioning the results of Fournet et al. (<https://doi.org/10.1371/journal.pone.0107878>):

“The general contact patterns did not appear to change significantly across seasons, as shown by the degree of similarity of the age-assortative mixing matrices and the results of the agent-based simulation which is in agreement with Fournet et al. [[16]](https://paperpile.com/c/HhccyM/KTOml).”

3. The authors could provide a very brief definition of empirical vs. synthetic contact matrices for the readers who are not familiar with the subject area.

Response: We thank the reviewer for the comment. We have modified the introduction to add more context to the differences between the two.

Methods:

4. What are the implications of using a convenience sample in terms of generalizability? The authors could compare some of the characteristics of this convenience sample with population characteristics based on national statistics (e.g., age structure, gender etc.)

Response: We thank the reviewer for the comment. Although we did not conduct a formal analysis, we addressed the point in the limitations section, stating that the convenience sample may only represent households within rural Haryana.

“As the survey was conducted among a convenience sample of households having a higher density of middle-aged (20-29) and old (60-69 years) individuals compared to the national statistics, the results are most representative of rural areas in Haryana, and may not generalize well to other areas.”

5. Was there a threshold number for defining “group contacts”? Initially, it was unclear whether the characteristics of each individual contact were recorded for group contacts. I suggest moving some of the information that is currently in the “age-assortative mixing matrices” earlier in the methods to improve clarity.

Response: We thank the reviewer for the comment. A group contact was defined as simultaneous encounter with more than one individuals. We moved this information earlier in the methods in the “Contact Diary Survey” subsection.

“In addition, the respondents were provided the option for reporting simultaneous encounters with more than one individual as "group" contacts, including the group size, duration of encounter, and the age range of individuals in the group. Details such as the age and gender of each individual in the group were not recorded. “

6. The duration of contacts is likely to be right-skewed. Taking the mean for each duration category assumes that the duration of contacts is uniformly distributed within each interval, which may not be the case. For example there may be a lot more contacts of duration 4-6 hours compared to 6-8 hours. If it is essential that a mean duration is computed, the authors should at least acknowledge the problem with this assumption.

Response: We thank the reviewer for the comment. We completely agree with the comment, however the data being recorded in a categorical scale led to the necessity to do so. We have added the following line in the limitations addressing this problem:

“The data on duration of contacts is categorical, and for the analysis we assume durations are equally probable within a category by calculating the mean of each category. In practice, it is more common to have contacts of a shorter duration (i.e, more 4-6 hour contacts than 6-8 hour contacts) and thus the durations are potentially right-skewed.”

7. The authors should justify the choice of a 5th degree polynomial. Does it have to do with the number of seasons examined, and does a higher degree polynomial run the risk of over-parameterizing?

Response: We thank the reviewer for the question. The choice was made based on a qualitative analysis of the polynomial fit to avoid overfitting the data and measuring evaluation metrics such as the Mean Squared Error, and the Mean Absolute Error. We have clarified the same in the Methods section.

“A polynomial regression model of degree 5 was fitted on the data, based on evaluation metrics like the Mean Squared Error and Mean Absolute Error. This model captured the complexity of the data, while still not being over-parameterized.”

Results:

8. Table 1: Columns should indicate whether the frequencies shown are the number of respondents or Responses.

Response: We thank the reviewer for the comment. We have clarified the following in the caption of *Table 1*:

“The numbers indicate the number of responses obtained, except for the ‘Total’ row in the ‘n’ column, which represents the total number of unique respondents who answered the survey.**”**

9. The directed graph is a useful way of visualizing the contact patterns in relation to age/gender. Is the thickness of the lines connecting the nodes proportional to the number of contacts between each pair of age-gender groups? If that is the case, it is surprising that the lines connecting the same age groups of different genders (e.g. 10-19M and 10-19F) have similar thickness to other pairs. Perhaps this could be adjusted to reflect level of contacts between groups?

Response: We thank the reviewer for the comment. The thickness of the lines is representative of the median number of contacts between each pair of age-gender categories. The network shows the median “individual” (and not the “total”) number of contacts. The reason to use individual contacts is the unavailability of gender-level information for group contacts. Our data shows that the median individual contacts between the 10-19M and 10-19F groups are similar to those with other groups. A large number of contacts between the two categories may happen in group settings (schools and playgrounds).

10. Given that the authors have chosen to use non-parametric methods for comparing all outcomes, it may be preferable to always display boxplots with medians rather than barplots and means.

Response: We thank the reviewer for the comment. We displayed boxplots and medians for the number and duration of contacts in Figures 1-4. In Figure 5, we intended to show the overall percentages and the total number of group contacts in different settings; therefore, we used bar plots to depict the same.

11. In general, the authors should include in all figure legends the total sample size making up each figure panel. E.g., for the seasonal comparisons, X respondents reported on Y contacts in at least 2 different seasons., etc.

Response: We thank the reviewer for the suggestion. Given the large number of figures and (already) long captions, we decided to forgo adding data to captions in cases where the data was already present in an appropriate table.

12. P-values should be reported consistently to the nearest 2 (or 3) decimal places unless it is a very small value (then should be reported as p<0.001).

Response: We thank the reviewer for the comment. We have made the changes in the manuscript as suggested.

13. In the section “Number of contacts” the authors mention “….but were not significantly different from all other age categories”. This seems to contradict with what is shown in Fig 1D-1F (any comparison that is not dark blue seems to indicate a significant difference).

Response: We thank the reviewer for the comment. We intended to convey the fact that while significantly different from some age categories, the age categories in question were not significantly different from every other age category. For example, there is no significant difference between 10-19 and 50-59 in winter, and thus, we cannot say with confidence that respondents aged 10-19 had the highest number of contacts.

14. Loss to follow up seemed to be substantial after phase one (Figure S1). The authors may want to comment on whether those lost-to-follow-up differed in terms of age/gender/number of contacts.

Response: We thank the reviewer for the comment. *Figure S1* already provides the number of individuals, households, and genders of individuals lost to follow-up. While analyzing the differences in contact patterns across seasons, we only consider respondents who are present in two or more seasons: We believe that this largely solves the problem, as the responses of individuals who only appeared in phase one would not be considered.

15. “Middle-aged people (20-39)” , and “Middle aged males (20-49 years): I suggest avoiding such phrasing. You can refer to the age category directly e.g., “males aged …” or “participants aged …” .

Response: We thank the reviewer for the suggestion. We have made these changes in the manuscript.

Discussion

16. One of the main findings is that there were more contacts in the winter and less in the monsoon season, though explanations of this finding are not sufficiently discussed.

Response: We thank the reviewer for the comment. As mentioned in the paper, there were more group contacts in winter when compared to any other season. The Contact Purposes section of the results was primarily made to investigate this, and we found several large group contacts occurring due to politics and weddings, which were rare in other seasons.

17. The authors should discuss whether the temporal effects are consistent spatially. How context-specific are these results in India, and do they have implications for other subnational or national areas?

Response: We thank the reviewer for the comment. The setting of this study was typical of a rural area of north India, and thus we believe that findings can be extrapolated to this context. However, India is a very diverse country, and due to differences in weather, social structures, and more, the findings may not be generalizable to urban populations or rural population of other regions. We believe the following line in the limitations addresses this:

“As the survey was conducted among a convenience sample of households having a higher density of middle-aged (20-29) and old (60-69 years) individuals compared to the national statistics, the results are most representative of rural areas in Haryana, and may not generalize well to other areas.”

18. The authors could make clear that the survey was conducted pre-COVID-19 pandemic, and that current contact patterns may be different.

Response: We thank the reviewer for the comment. We have added the following line in the Limitations section to address this:

“The survey data was also gathered before the COVID-19 pandemic, which might have altered the contact patterns in present-day society”

19. School closures were not considered in the analysis, which is relevant for the most important nodes (age groups 10-19). This is likely to impact contact patterns, and it may be a good idea to include in the supplement an analysis (comparing “during school closures” vs. “during school term-time”) and/or in the discussion.

Response: We thank the reviewer for the constructive suggestion. Unfortunately, our data does not cover periods when schools were closed. We could only analyze the three seasons for which we had collected data.

20. Have the authors considered looking at both age and gender assortativity? Only age assortativity is presented (and stratified by gender).

Response: We thank the reviewer for the comment. We considered performing several such additional analyses, but doing so would massively increase the size of our manuscript. As such, our current write-up is focused on age assortativity with gender stratification.

Supplementary material

21. The supplement could benefit from the addition of the questionnaire used for recording contacts.

Response: We thank the reviewer for the response. We have added the questionnaire into the supplementary material.

22. The authors should cite all supplementary figures and tables in the main manuscript.

Response: We thank the reviewer for the comment. We have cited Supplementary *Figure S5* in the ‘Age Assortativity in Contacts’ section of the Results.

23. Please revise all supplementary figures as many of them contain spelling errors and errors in the axes names.

Response: We thank the reviewer for the comment. We have incorporated the changes in the figures as per suggestion.
