# Supplementary material for: Seasonal variations in social contact patterns in a rural population in north India: Implications for pandemic control
Source: PLoS One. 2024 Feb 22;19(2):e0296483. doi: 10.1371/journal.pone.0296483 (PMC10883557; doi:10.1371/journal.pone.0296483)
Supplement: Attachment — Submitted filename: Response to Reviewers.docx [file pone.0296483.s021.docx]

We thank the Editor for bringing an incomplete ethics statement to our attention.

We have now changed the lines:

“We received written consent from all participants over 7 years of age. Caregivers provided written consent for participants below 7 years of age.”

To

“We received written consent from all participants 18 years of age and above. All those between 7 years and 18 years provided written assent in addition to the written consent of the caregivers. Caregivers provided written consent for participants below 7 years of age.”

This reflects what was actually done. The earlier version was incomplete.
